# The *Drosophila *methyl-DNA binding protein MBD2/3 interacts with the NuRD complex via p55 and MI-2

**DOI:** 10.1186/1471-2199-5-20

**Published:** 2004-10-29

**Authors:** Joachim Marhold, Alexander Brehm, Katja Kramer

**Affiliations:** 1 Research Group Epigenetics, Deutsches Krebsforschungszentrum, Im Neuenheimer Feld 580, 69120 Heidelberg, Germany; 2 Adolf-Butenandt-Institut, Ludwig-Maximilians-Universität, Schillerstrasse 44, 80336 München, Germany

## Abstract

**Background:**

Methyl-DNA binding proteins help to translate epigenetic information encoded by DNA methylation into covalent histone modifications. *MBD2/3 *is the only candidate gene in the *Drosophila *genome with extended homologies to mammalian MBD2 and MBD3 proteins, which represent a co-repressor and an integral component of the Nucleosome Remodelling and Deacetylase (NuRD) complex, respectively. An association of *Drosophila *MBD2/3 with the *Drosophila *NuRD complex has been suggested previously. We have now analyzed the molecular interactions between MBD2/3 and the NuRD complex in greater detail.

**Results:**

The two MBD2/3 isoforms precisely cofractionated with NuRD proteins during gel filtration of extracts derived from early and late embryos. In addition, we demonstrate that MBD2/3 forms multimers, and engages in specific interactions with the p55 and MI-2 subunits of the *Drosophila *NuRD complex.

**Conclusion:**

Our data provide novel insights into the association between *Drosophila *MBD2/3 and NuRD proteins. Additionally, this work provides a first analysis of the architecture of the *Drosophila *NuRD complex.

## Background

Methyl-DNA binding proteins are connecting DNA methylation to transcriptional silencing [1-4]. Up to now, six methyl-DNA binding proteins could be identified in vertebrates [5]. MeCP2, MBD2 and MBD3 can be found in large chromatin complexes containing histone deacetylase activity [1,6,4,3] whereas MBD4 is involved in DNA mismatch-repair [7]. MBD1 has been shown to repress transcription in cell culture [8] and recruits the histone H3-K9 methyltransferase SETDB1 to the chromatin assembly factor CAF-1 during S phase [9]. MBD2, which can bind methylated DNA [6], is a transcriptional repressor recruiting a Nucleosome Remodelling and Deacetylase complex (NuRD) to methylated CpG dinucleotides [6,3], whereas mammalian MBD3, which is not able to bind methylated DNA [10] is an integral component of NuRD [3]. Kaiso, a transcriptional repressor protein, can bind directly to CpG methylated DNA even though it lacks a conserved methyl-DNA binding domain [11]. Kaiso is a component of a subpopulation of MeCP1 complexes that lack MBD2 [11].

The *Drosophila *gene *MBD2/3 *encodes a protein, which shares high homology to mammalian MBD2 and MBD3 [12,4]. Due to differential splicing, *Drosophila MBD2/3 *is expressed in two isoforms, the smaller one is lacking part of the putative methyl-DNA binding domain [12-15]. The large isoform is expressed during early development, whereas the small isoform can only be detected during late embryogenesis [14,15]. In insect cells expressing only the small MBD2/3 isoform, this protein was found to be associated with components of the *Drosophila *NuRD complex [12-14]. Moreover, this protein could repress transcription effectively in transfected *Drosophila *cells [13,14].

The NuRD complexes of vertebrates are a heterogeneous group of complexes containing both histone deacetylase and nucleosome remodelling activities [3]. NuRD complexes comprise at least seven proteins. The ATP- dependent nucleosome-remodelling activity is mediated by MI-2, which contains a SWI2/SNF2-type helicase/ATPase domain, two chromodomains and two PHD fingers [16]. The related MTA1, MTA2 and MTA3 proteins have been found in various complex preparations [17,4,3,18]. MTA1 was originally identified as being overexpressed in metastatic carcinomas [19]. The histone deacetylases HDAC1 and HDAC2 and the two histone binding proteins RbAp46 and RbAp48 form the histone deacetylase core of NuRD complexes. Finally, as mentioned above, mammalian MBD3 is an integral component of at least some NuRD complexes [3].

Strikingly, the *Drosophila *genome contains clear homologues for all verterbrate NuRD proteins. Recombinant *Drosophila *MI-2 was shown to have ATPase and nucleosome mobilization properties [20]. In *Drosophila *the HDAC gene *Rpd3 *is important for segmentation of the embryo [21]. *Drosophila *p55, a WD-40 protein, is homologous to the histone deacetylase-associated proteins RbAp46 and RbAp48 [22]. Finally, *Drosophila *MTA-like displays extensive homology to the vertebrate MTA proteins [23].

The strong conservation between vertebrate NuRD complexes and the *Drosophila *NuRD complex implies a conserved function during the animal development. In previous studies cell lines were analysed that express only the small isoform of MBD2/3, lacking part of the putative methyl-DNA binding domain and a *Drosophila *specific domain. In order to analyse isoform-specific differences of MBD2/3 and their ability to bind NuRD proteins, we now extend our analysis to both isoforms in *Drosophila *embryos.

## Results

### MBD2/3 is associated with *Drosophila *NuRD proteins

**Figure 1:**
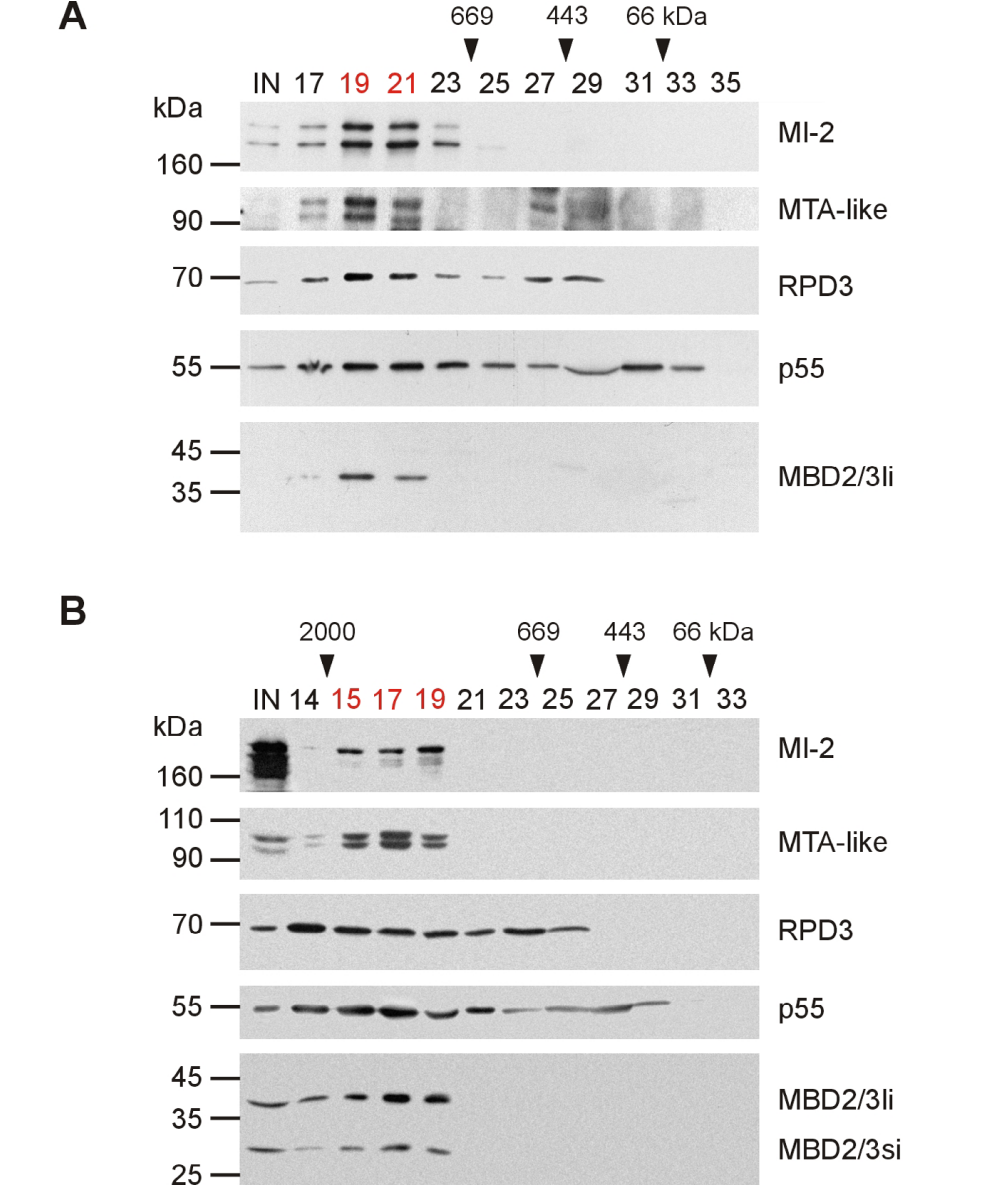
**Cofractionation of *Drosophila *NuRD homologues in embryonic protein extracts **Nuclear protein extracts were prepared from (A) 2–4 h and (B) 0–12 h old embryos, respectively and size-fractionated by FPLC using a Superose 6 column. Proteins from selected fractions were then separated by SDS gel electrophoresis and analysed by Western blotting. (A) During early embryogenesis the long isoform of MBD2/3 (MBD2/3li) cofractionated with MI-2 and MTA-like in fractions 19 to 21 (indicated in red), which correspond to a molecular weight of approx. 1 MDa. The small isoform of MBD2/3 is not expressed at this stage of embryogenesis. (B) During later stages of embryogenesis both isoforms of MBD2/3 cofractionated with MI-2 and MTA-like in fractions 15 to 19 (indicated in red), which correspond to a molecular weight of approx. 2 MDa. The size of marker proteins is shown left and on top, IN indicates input protein.

An association of *Drosophila *MBD2/3 with the NuRD complex has been suggested previously [14,12]. However, these experiments were done in vitro or with protein extracts from tissue culture cells that express only the small isoform of MBD2/3. In addition, only a limited number of NuRD proteins were analysed. To confirm the association between MBD2/3 and NuRD in *Drosophila *embryos we size-fractionated nuclear protein extracts from 2–4 h or 0–12 h wild type embryos, respectively, by Superose 6 gel filtration chromatography. Proteins from individual fractions were then separated by standard SDS-PAGE and analysed by Western blotting using antibodies directed against known homologous subunits of the NuRD complex. MI-2 and MTA-like, which are homologues of subunits that are specific for vertebrate NuRD complexes [24,3] cofractionated with an apparent molecular weight of 1 MDa when extracts derived from early embryos (2–4 h) were applied (Fig. 1A, fractions 19 to 21). When extracts of late embryos (0–12 h) were analysed, MI-2 and MTA-like were detected in fractions 15 to 19, which correspond to an apparent molecular weight of approx. 2 MDa (Fig. 1B). This result indicates that the formation of NuRD is developmentally regulated during embryogenesis. Additionally, we found a precise cofractionation of the large isoform of MBD2/3 (Fig. 1, MBD2/3li) with MI-2 and MTA-like in both cases. The small MBD2/3 isoform (Fig. 1B, MBD2/3si) present in extracts derived from late embryos likewise cofractionated with MI-2 and MTA-like. This suggests a close association of both MBD2/3 isoforms with the *Drosophila *NuRD complex. The RPD3 and p55 proteins also cofractionated with MBD2/3 isoforms, but they were also found in fractions of lower molecular weight (Fig. 1). This is in agreement with the fact that both RPD3 and p55 are also components of other chromatin related complexes [25,26].

### Interactions between MBD2/3 and NuRD homologues

**Figure 2:**
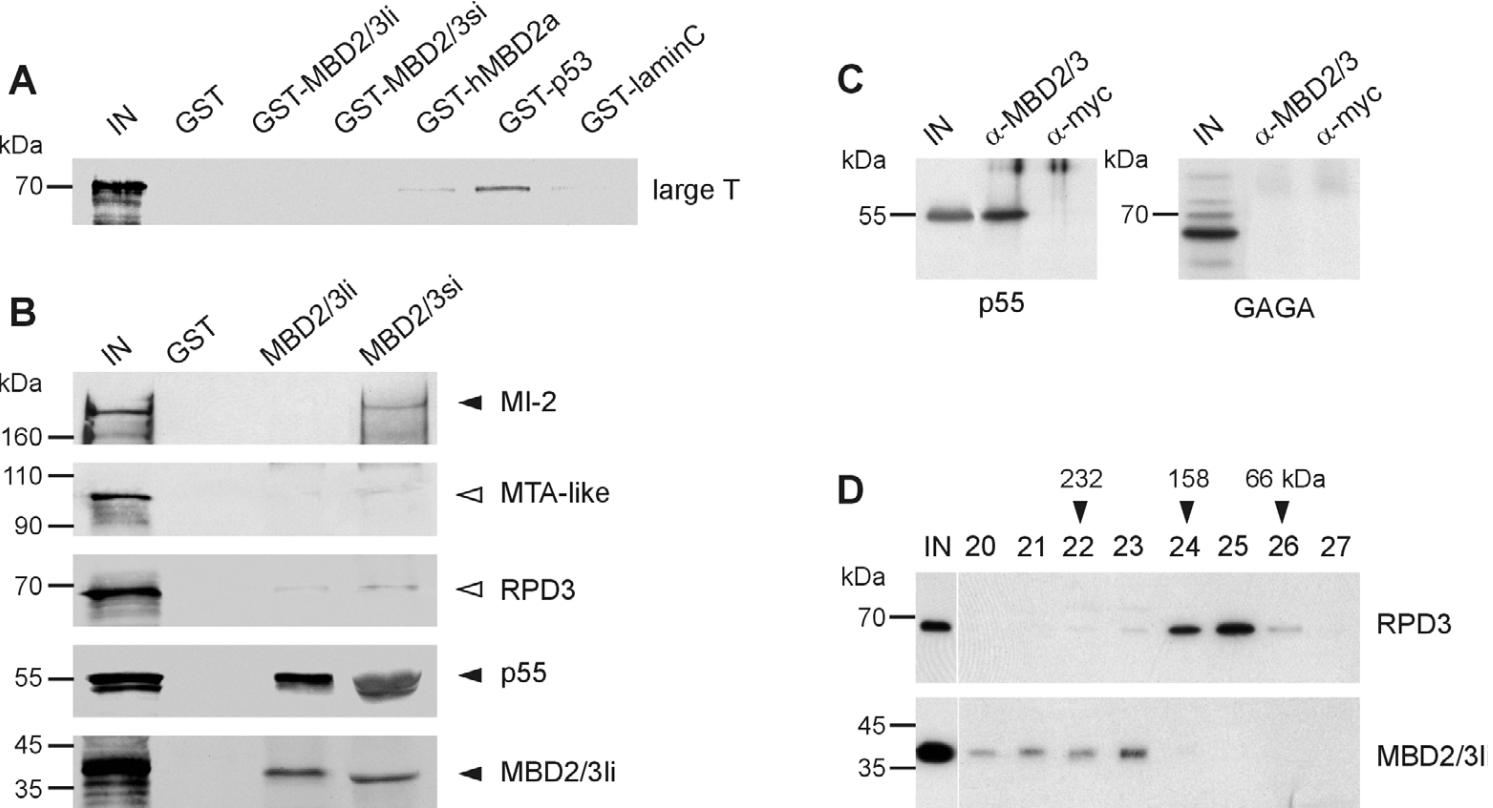
**Interactions between MBD2/3 and NuRD homologues in a GST-pulldown assay **(A) Analysis of interactions between radioactively labelled SV40 large T antigen (large T) and a number of control GST fusion proteins under stringent buffer conditions. Significant amounts of SV40 large T were only precipitated by p53. The faint bands seen with some of the other proteins were considered background. (B) GST-MBD2/3 fusion proteins for long and small isoforms (MBD2/3li, MBD2/3si, respectively) efficiently precipitated radioactively labelled MBD2/3 long isoform and p55 (solid arrowhead). Similarly, MI-2 protein could be precipitated by the small GST-MBD2/3 isoform (solid arrowhead). No interaction could be observed between long MBD2/3 or small MBD2/3 isoforms and MTA-like or RPD3 (open arrowheads). (C) A MBD2/3-specific antibody immunoprecipitates p55, but not GAGA factor from embryonic nuclear extracts. No proteins were detectable in control immunoprecipitations with a myc-specific antibody. (D) Size-fractionation of baculovirus-expressed MBD2/3 long isoform and RPD3. Baculovirus-expressed MBD2/3li elutes in fractions that significantly exceed the calculated molecular weight of the protein (36 kDa), thus indicating that MBD2/3li efficiently multimerizes in solution. This effect appeared to be specific for MBD2/3 and was not observed with baculovirus-expressed RPD3 protein (58kDa). The size of marker proteins is shown left and on top, IN indicates input protein.

To analyse the association between MBD2/3 and the NuRD complex in more detail and to identify direct NuRD interaction partner(s) of MBD2/3 we first used a GST-pulldown assay. In order to eliminate unspecific interactions with NuRD proteins, we established highly stringent assay conditions. This was achieved by assessing the interactions between radioactively labeled SV40 large T antigen (Fig. 2A, large T) and a number of control GST fusion proteins. When we used an incubation and washing buffer with high salt and detergent concentrations, significant amounts of SV40 large T were precipitated by p53 only (Fig. 2A), which is known to be a strong interactor of the large T antigen [27]. Residual binding detected with some of the other proteins (Fig. 2A) was considered background.

We then performed the MBD2/3-NuRD interaction assay using these conditions and observed strong interactions only between the long and small MBD2/3 isoforms (Fig. 2B, MBD2/3li and MBD2/3si, respectively) and p55. To confirm the association between MBD2/3 and p55 in *Drosophila *embryos we immunoprecipitated nuclear extracts with MBD2/3-specific antibodies and with myc-specific control antibodies. Precipitates were analysed by Western blot for the presence of p55 and GAGA factor. This revealed a specific association of MBD2/3 with the *Drosophila *NuRD homologue p55, but not with the unrelated GAGA factor (Fig. 2C).

A weaker interaction could also be detected between the small MBD2/3 isoform and MI-2 (Fig. 2B). Neither RPD3 nor MTA-like binding exceeded the background level defined by our pilot experiment with SV40 large T protein (Fig. 2B). Our results thus identified p55 and MI-2 as the direct interaction partners of MBD2/3 in the NuRD complex. In addition, our results from the GST-pulldown assay indicate that *Drosophila *MBD2/3 forms dimers or multimers, similar to mammalian MBD2 and MBD3 [28]. To discriminate between the latter two possibilities we expressed a recombinant long MBD2/3 isoform and RPD3 in insect cells using a baculovirus expression system. Extracts from infected cells were subjected to Superdex 200 gel filtration and fractions were analysed by Western blot. The 58 kDa RPD3 protein eluted in fractions corresponding to molecular weights ranging from 66 kDa to 158 kDa, which suggested that RPD3 exists as monomers or dimers in solution (Fig. 2D). In contrast, the 36 kDa MBD2/3 isoform showed a strikingly different elution profile (Fig. 2D). The long MBD2/3 isoform eluted in a broad peak ranging from more than 158 kDa up to high molecular weight fractions (>400 kDa). No MBD2/3 was detected in fractions corresponding to the expected size of MBD2/3 monomers or dimers. This result is consistent with our earlier observation that MBD2/3 forms distinct aggregates in embryonic nuclei [15] and suggests that the protein efficiently oligomerises to form high-molecular weight complexes in solution.

**Figure 3:**
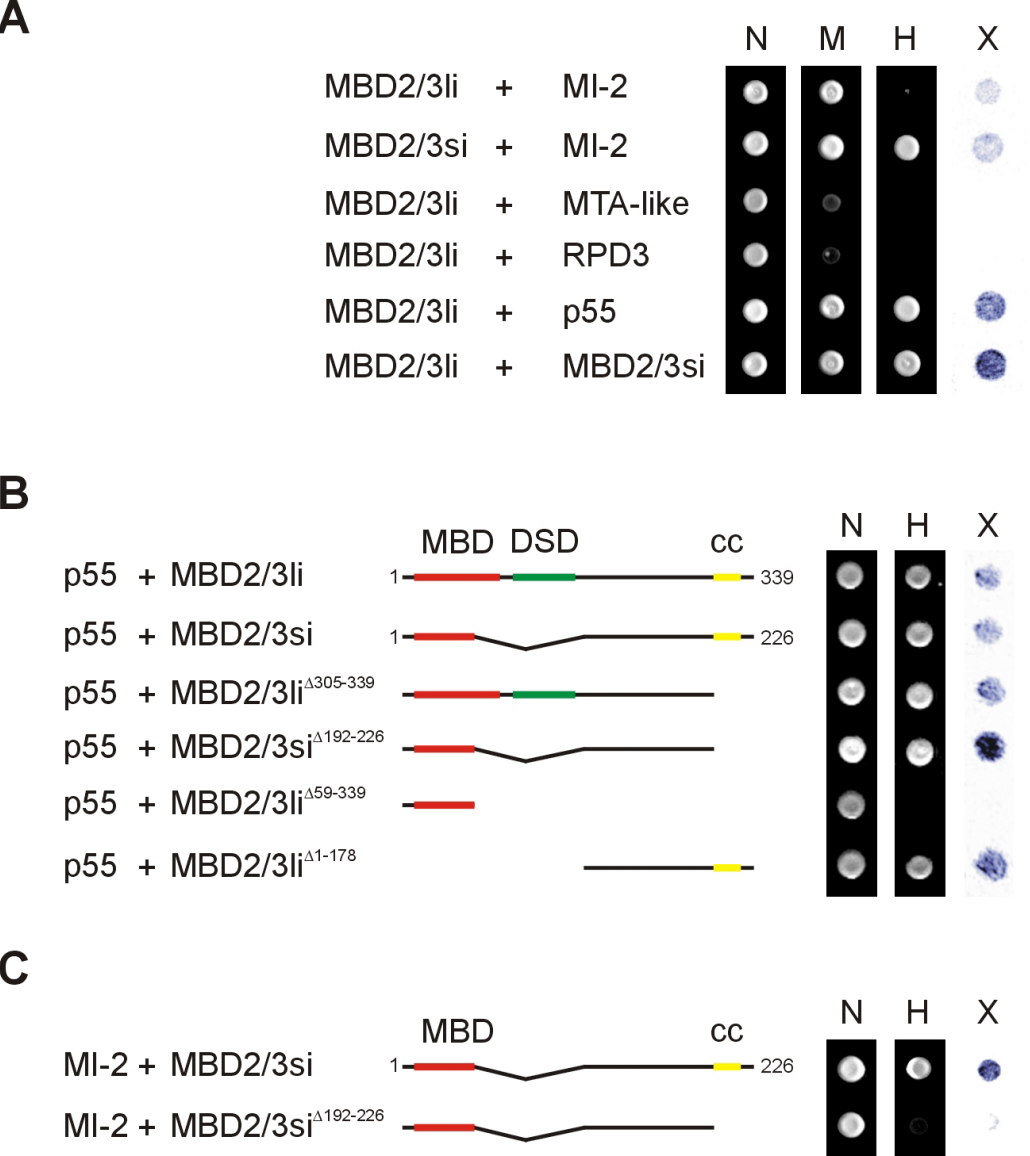
**Interactions between MBD2/3 and NuRD homologues in a yeast two-hybrid assay **(A) An MBD2/3 long isoform bait efficiently transactivated reporter gene transcription in yeast expressing p55 or MBD2/3 small isoform prey constructs, respectively. A weaker transactivation could be seen upon expression of a MI-2 prey construct (panel M). An MBD2/3 small isoform bait transactivated reporter gene transcription co-expressing a MI-2 prey also under high-stringent conditions (panel H). No transactivation could be observed with MTA-like or RPD3 preys. N shows growth on non-selective plates, M on medium-stringency plates and H on high-stringency plates. X indicates X-gal staining of colonies grown on non-selective plates. (B and C) Delineation of interaction domains in a yeast two-hybrid assay. The methyl-DNA binding domain (MBD) is highlighted in red, the *Drosophila *specific domain (DSD) in green and the C-terminal coiled-coil domain (cc) in yellow. (B) The C-terminal region of MBD2/3 interacts with p55. The full-length p55 construct was used as a bait and various deletion mutants of MBD2/3 were used as preys. This identified the region between amino acids 178 and 305 of the MBD2/3 long isoform as the p55 interaction domain. (C) Deletion of the coiled-coil domain abolishes interactions between MBD2/3 small isoform and MI-2 under high stringent conditions.

To confirm these interactions in an independent set of experiments we also analysed the association between MBD2/3 isoforms and the NuRD proteins in a yeast two-hybrid assay. In agreement with our previous results, growth on highly selective (Fig. 3A, panel H) plates was only observed upon co-expression of the MBD2/3 small isoform and either MI-2, MBD2/3 long isoform or p55, and upon co-expression of both MBD2/3 isoforms. These interactions were confirmed by X-Gal staining of filter-lifted yeast colonies (Fig. 3A, panel X). In these assays the two MBD2/3 isoforms differed only with regard to MI-2 binding (Fig. 3A). However, differential binding could be eliminated by a slight reduction in the stringency of the assay (Fig. 3A, panel M), which suggested that both isoforms of MBD2/3 are capable of interacting with MI-2. Consistent with our GST-pulldown assays no interaction could be detected between the MBD2/3 long isoform and RPD3 or MTA-like (Fig. 3A).

Next we sought to delineate the domains that mediate the association between the MBD2/3 isoforms and their interacting proteins. To this end, we generated several MBD2/3 deletion mutants and tested their interaction with other proteins in a yeast two-hybrid assay. In a first series of experiments we tested the MBD2/3 mutants for their ability to interact with p55. This identified the region between residues 178 and 305 of the MBD2/3 long isoform as the p55 interaction domain (Fig. 3B). We also delineated the domain that mediates the interaction with MI-2. Our experiments revealed a strongly reduced interaction between a MBD2/3 small isoform derivative lacking the coiled-coil domain and MI-2 (Fig. 3C). It has been shown previously that vertebrate MBD2 and MBD3 form homo- and heterodimers via their N-terminal MBD domain and their C-terminal coiled-coil like sequences [28]. Our findings might reflect a direct interaction between the coiled-coil domain and MI-2 or a requirement for efficient MBD2/3 multimerization for MI-2 interaction. In conclusion, our results identify distinct regions in the MBD2/3 protein that mediate protein-protein interactions with other NuRD proteins.

**Figure 4:**
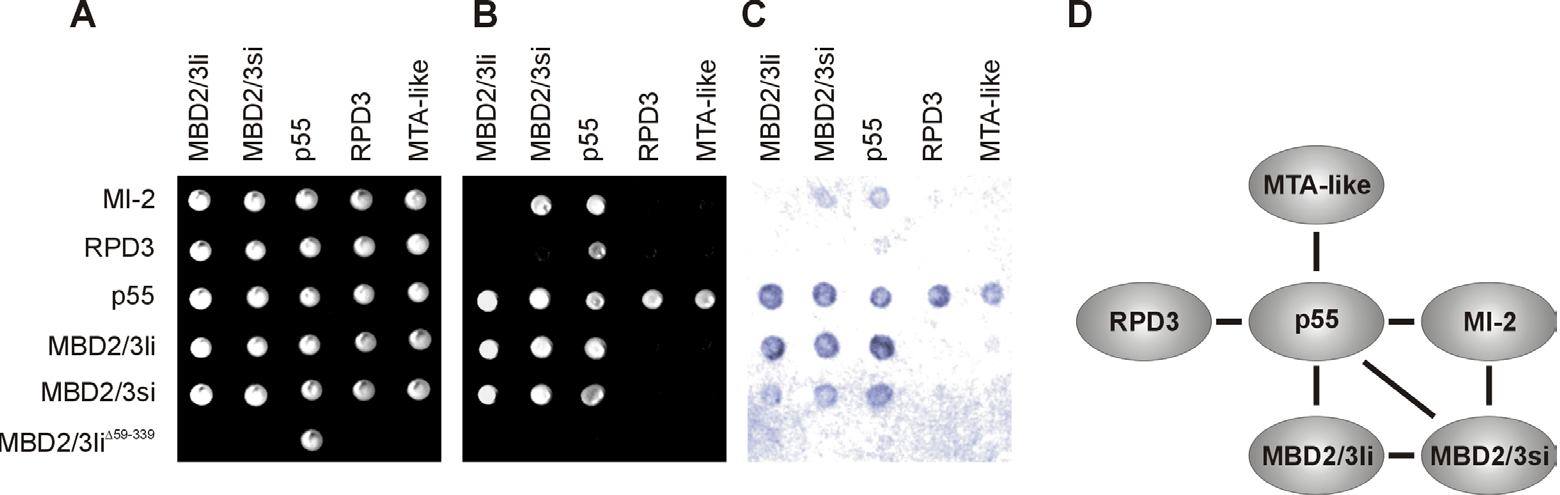
**Analysis of the architecure of NuRD **(A) Yeast colonies growing on non-selective plates carrying bait (left panel) and prey (top panel) NuRD and MBD2/3 constructs. (B) Replica plate with high-selective medium. Only yeast colonies with constructs encoding interacting proteins are able to grow. MBD2/3li^Δ59-339 ^represents a truncated MBD2/3li construct that was used to confirm the specificity of the interaction between p55 and MBD2/3. (C) X-Gal staining of yeast colonies to confirm interactions. (D) Schematic illustration of protein-protein interactions in the NuRD complex. Homodimerization of p55 and MBD2/3 are not shown.

In order to analyse the architecture of the NuRD complex, we performed a detailed yeast two-hybrid analysis including additional bait and prey constructs for NuRD proteins. Bait constructs for MI-2, RPD3, p55 and both MBD2/3 isoforms and prey constructs for MTA-like, RPD3, p55, and both MBD2/3 isoforms were co-transformed in all possible combinations and transformation was confirmed by growth on non-selective plates (Fig. 4A). Colonies were then replicated onto highly selective plates that allow growth only upon interaction between the two expressed proteins (Fig. 4B). Finally, we performed a filter lift assay followed by X-Gal staining to confirm the interactions (Fig. 4C). In addition to the results presented above, we found strong interactions between p55 and all NuRD proteins. We also observed a homotypic interaction for p55 but no homotypic interaction for RPD3. To confirm the specificity of the interactions, we tested most of the constructs reciprocally by changing the bait and prey vectors for expression of NuRD proteins and MBD2/3 isoforms. This data allows the establishment of a more detailed model of the NuRD complex. Fig. 4D summarizes the data from all experiments. p55 interacted with MI-2, MTA-like, RPD3, and both MBD2/3 isoforms. The small isoform of MBD2/3 interacted with MI-2 as well as with the large MBD2/3 isoform. Homodimerization of p55 and MBD2/3 is not shown in the model.

## Discussion

It has been previously suggested that MBD2/3 is associated with the *Drosophila *NuRD complex [14]. This study determined that the small isoform MBD2/3 coelutes with some putative *Drosophila *NuRD subunits during fractionation of extracts derived from a *Drosophila *cell line. We have now extended this analysis to show that both isoforms of MBD2/3 coelute with NuRD homologues during fractionation of embryonic extracts. This data provides further evidence for a direct interaction between MBD2/3 and the NuRD complex.

Using several independent assays, we have demonstrated that MBD2/3 engages in homotypic interactions to form multimers. This effect is consistent with the formation of foci in embryonic nuclei [15] and also reminiscent of the interactions described for vertebrate MBD2 and MBD3 [28].

In addition, our data provides new insights into the association between MBD2/3 and NuRD. For example, we have shown that p55 appears to be the primary interaction partner of MBD2/3. We also observed a strong interaction between the small isoform MBD2/3 and MI-2, but not between MBD2/3 small isoform and MTA-like or RPD3. Additionally, we found interactions between p55 and all NuRD proteins, as well as a p55 homotypic interaction. The last finding is consistent with the fact that in the vertebrate NuRD complex the two p55 homologues RbAp46 and RbAp48 were identified as integral components [3]. We note that the vertebrate NuRD complex also contains the two RPD3 homologues HDAC1 and HDAC2 [3]; unexpectedly, we were not able to detect any homotypic interaction of RPD3 in the yeast two-hybrid assay. One explanation could be that the dose of the two co-expressed RPD3 bait and prey proteins could interfere with the reporter gene activity due to their inherent histone deacetylation activity. However, the gel filtration assay revealed that the 58 kDa RPD3 protein eluted in fractions corresponding to molecular weights ranging from 66 kDa to 158 kDa, which suggested that RPD3 can interact homotypically (Fig. 3C).

It is possible that more complex interactions are involved in the assembly of the NuRD complex but they might not have been detectable under the stringent conditions of our assays. The interaction between MBD2/3 and MI-2 detected in our assays could also contribute to the specific association between MBD2/3 and the NuRD complex.

The interaction between MBD2/3 and p55 could promote the assembly of specialized chromatin structures in the fly. p55 is a WD-40 repeat protein that is involved in many aspects of chromatin organization [29]. For example, p55 has been shown to be a component of the *Drosophila *CAF-1 complex that promotes nucleosome assembly [22]. In addition, p55 is also contained in the NURF chromatin remodelling complex [30] and in the E(Z) complex that regulates homeotic gene expression [25,26]. A mutant *Drosophila *allele for *p55 *is not available, but results obtained from *Arabidopsis *mutants with decreased levels of a p55 homologue indicate that the protein plays an important role in stabilizing epigenetic chromatin structures [31].

## Conclusions

The goal of this study was to identify the interacting partners of the *Drosophila *methyl-DNA binding protein MBD2/3 within the *Drosophila *NuRD complex. We identified p55 and MI-2 as the primary interacting partners. We also found homo- and heterotypic interactions of the MBD2/3 isoforms, similar to vertebrate MBD2 and MBD3. Additionally, yeast two-hybrid assays revealed that p55 is able to specifically interact with all other NuRD proteins and can form homotypic interactions. Our data provides for the first time information about the architecture of the *Drosophila *NuRD complex. This allows us to develop a structural model of the NuRD complex.

## Methods

### DNA constructs

Constructs for the yeast two-hybrid assay were generated by PCR amplification from cDNA clones [32] using specific primers (Tab. 1) with an attached restriction endonuclease target site for cloning. The PCR products were subsequently cloned into pGBKT7 or pGADT7 (Clontech) using standard procedures. All constructs were sequenced and in vitro translated to confirm the expression of corresponding proteins. Additionally, the bait constructs were expressed in yeast and expression of the fusion proteins was confirmed by Western analysis.

### Antibodies

The following antibodies have been described previously: rabbit anti-MI-2 [20], rabbit anti-MTA1 [19,4], rabbit anti-RPD3 [20], rabbit anti-p55 [22], rabbit anti-MBD2/3 [14] and rabbit anti-GAGA [33].

### Immunoprecipitations

Immunoprecipitations were caried out in 300 mM KCl supplemented with 0.2 % NP-40. Rabbit anti-MBD2/3 or mouse anti-myc (Clontech) antibodies were added to 75 μl of nuclear extracts prepared as mentioned below and incubated for 12 h at 4°C. Protein G beads (Amersham) were blocked in 3 mg/ml BSA for 20 min at room temperature, washed three times with 300 mM KCl, 0.2 % NP-40 and added to the samples. Incubation was carried out for an additional 1 h at 4°C. The beads were collected by centrifugation and washed four times in 1 ml 300 mM KCl, 0.2 % NP-40. The beads were resuspended in loading buffer for SDS-PAGE and vigorously vortexed for 15 sec. The immunoprecipitates were separated by SDS-PAGE, without futher boiling, transferred to a PVDF membrane and probed with antibodies against RPD3, p55 and GAGA factor, respectively.

### Preparation of protein extracts and gel filtration

For baculovirus expression, the MBD2/3 cDNA [32] was subcloned into the pVL1392 baculovirus transfer vector. Transfer vector and linearised baculovirus DNA were cotransfected into Sf9 cells using the Bac'n'Blue transfection kit (Invitrogen) and recombinant virus was amplified according to the manufacturer's instructions. Whole cell extracts of infected Sf9 cells were generated by resuspending cell pellets in lysis buffer (20 mM Hepes pH 7.6, 200 mM KCl, 0.1 % NP40), incubation on ice for 10 min, three freeze/thaw cycles and sonication. Nuclear extracts from *Drosophila *embryos were prepared as described previously [20]. Extracts were cleared by centrifugation and passaged through a 0.2 μm filter. 200 μl of cleared extract was applied to Superdex 200 HR 10/30 or Superose 6 HR 10/30 gel filtration columns (Amersham Pharmacia) and resolved in 20 mM Hepes pH 7.6 and 300 mM KCl on an Äkta Purifier system (Amersham Pharmacia) according to the manufacturer's instructions.

### GST-pulldown assays

^35^S-methionine-labelled proteins were generated by in vitro trancription/translation of pGADT7_T, pGADT7_Mi-2, pGADT7_MTA-like, pGADT7_Rpd3, pGADT7_p55, pGADT7_MBD2/3 long isoform and pGADT7_MBD2/3 small isoform using the TNT coupled reticulocyte lysate system (Promega) according to the manufacturer's protocol. GST-MBD2/3 long isoform and GST-MBD2/3 small isoform fusionproteins were obtained by cloning the coding region of the two isoforms in pGEX4T1 (Amersham) and subsequent expressing of the constructs in BL-21 bacteria according to the manufacturer's protocol. GST-MBD2a was obtained from Hidetoshi Fujita [34]. GST-pull downs were performed under the following conditions: As incubation and washing buffer we used 20 mM HEPES, pH 7.8, 300 mM NaCl, 0.1 % desoxycholate, 0.1 % IGEPAL, 10 % glycerol. The radioactively labelled proteins were incubated in incubation buffer for 3 h at room temperature with GST fusion proteins coupled to Sepharose 4B (Amersham) or with GST alone coupled to Sepharose 4B. Beads were then washed five times for 10 min at room temperature. After the last washing step, beads were boiled in SDS loading buffer for 10 min and loaded onto standard SDS-polyacrylamide gels. After separation by SDS-PAGE, proteins were blotted onto a PVDF membrane, which was stained with Ponceau S, dried and exposed on X-ray films.

### Yeast two-hybrid assays

The Matchmaker Two-Hybrid system 3 (Clontech) was used for all experiments, according to the manufacturer's instructions. The AH109 strain was used as host and transformed with various constructs (see above) using standard procedures. Transformants were selected on SD/-Ade/-His/-Leu/-Trp plates.

## Authors' contributions

KK and JM carried out the molecular interaction studies. AB carried out the gel filtration experiments. JM conceived of the study, and participated in its design and coordination. All authors read and approved the final manuscript.

**Table 1:** PCR primers used for the cloning of recombinant plasmids

MBD-BD-F: gga att cat gca aat gaa ccc gag cgt c
MBD-BD-R: tcc ccc ggg tgt ctt gag tgc atc ctg cag
Mi2-BD-F: cgc cat atg atg gca tcg gag gaa gag aat gac
Mi2-BD-R: gcg gcc tcc atg gcc gac gcc gga att att cga tag c
MTA-AD-F: ccg gaa ttc atg gcc aca aat atg tat cga gtc gg
MTA-AD-R: tcc ggg ccc ggt gac act ata gaa ctc gag
Rpd3-BD-F: atg gcc atg gat gca gtc tca aca gc
Rpd3-BD-R: ggc cgc tgc aga atg ttg ttc tcc ttg gcg
Rpd3-AD-F: cgc tca tat gat gca gtc tca cag c
Rpd3-AD-R: gca gct cga gaa tgt tgt tct cct tgg cg
p55-BD-R: ggc tca atc ttt ggt tat ggc gaa ttg gat ccg cg
p55-AD-F: ccg gaa ttc atg gtg gat cgc agc g
p55-AD-R: ggc gag ctc tta agc ggt att ggt ttc taa ctc gg
MBD-AD-F: gga att cat gca aat gaa ccc gag cgt c
MBD-AD-R: ccg ctc gag tgt ctt gag tgc atc ctg cag
MBD-AD-Δ59-339: ccg ctc gag cca cct tgt tat tgt tgt tgt tgc

BD designates primers used for the binding domain-constructs, AD designates primers used for the activation domain-constructs. F indicates forward primers, R indicates reverse primers.
